# Supplementary material for: A whole-animal phenotypic drug screen identifies suppressors of atherogenic lipoproteins
Source: eLife. 2026 Aug 27;14:RP105314. doi: 10.7554/eLife.105314 (PMC13521566; doi:10.7554/eLife.105314)
Supplement: Supplementary file 2 [file elife-105314-code1.zip › JHDL_primary_result/Prochlorperazine dimaleate/Prochlorperazine dimaleate_JHDL_31_009_004_2020_01_17_10_36.pdf]

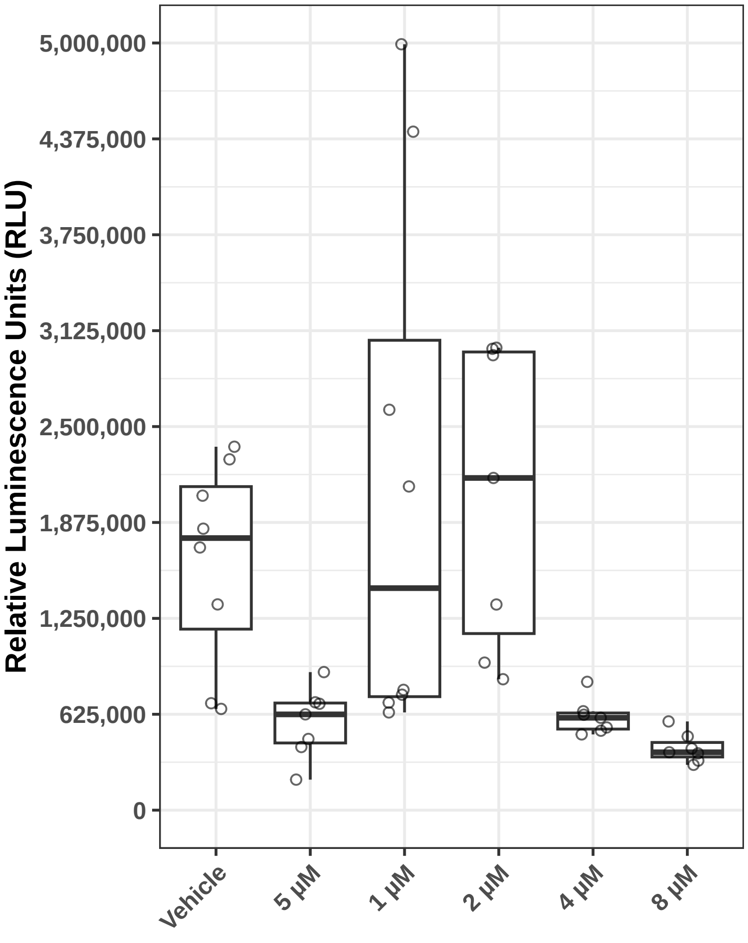

Relative Luminescence Units (RLU)
5,000,000
4,375,000
3,750,000
3,125,000
2,500,000
1,875,000
1,250,000
625,000
0
Vehicle
5 μM
1 μM
2 μM
4 μM
8 μM
